# Functional profiling of the rhizospheric Exiguobacterium sp. for dimethoate degradation, PGPR activity, biofilm development, and ecotoxicological risk

**DOI:** 10.1038/s41598-024-80559-z

**Published:** 2024-11-26

**Authors:** Saheli Sur, Mythili Sathiavelu

**Affiliations:** School of Biosciences and Technology, Vellore Institute of Technology, Vellore, Tamil Nadu India

**Keywords:** Biodegradation, Bacteria, Pesticides, Plant-growth promoting bacteria, Ecotoxicity, Microbiology, Environmental sciences

## Abstract

This study introduces an indigenous bacterial strain, *Exiguobacterium* sp. (L.O), isolated from sugarcane fields in Sevur, Tamil Nadu, which has adapted to prolonged exposure to dimethoate. The strain demonstrated the capability to utilize 150 ppm of dimethoate as its sole carbon source, achieving a remarkable degradation rate of 95.87% within 5 days in mineral salt media. Gas chromatography–mass spectrometry (GC–MS) analyses identified the presence of intermediate by-products formed during degradation, like methyl diethanol amine and aspartyl glycine ethyl ester. Notably, phosphorothioic O, O, S-acid, an expected end product in the degradation of dimethoate, was also identified, further confirming the strain’s effective metabolic breakdown of the pesticide. Further degradation study and analysis of changes in functional group was performed by FTIR, and a hypothetical degradation pathway was elucidated showing the course of dimethoate metabolism by the strain. *Exiguobacterium* sp. (L.O) also displayed significant plant growth-promoting traits, including the production of HCN, IAA, and ammonia and the formation of biofilms, which enhance its utility in agricultural applications. The ecotoxicity study revealed the degradation by-products exhibited reduced toxicity compared to the parent compound dimethoate, highlighting the strain’s potential not only for bioremediation but also for supporting sustainable agricultural practices. This research presents a novel application of *Exiguobacterium* sp. (L.O), integrating the bioremediation of the organophosphate pesticide dimethoate with agricultural enhancement. This approach is critical for addressing the challenges associated with pesticide pollution in agricultural practices. This study is likely the first to demonstrate the application of this strain in the degradation of dimethoate, as suggested by an extensive review of the literature.

## Introduction

A surge in the human population to more than 9 billion by the year 2050 parallelly demands an urgent rise in crop production by 60%^1^. This urgent demand has led to the widespread adoption of intensive agricultural practices, which often rely heavily on agrochemicals such as fertilizers and pesticides. While these practices have elevated crop yields, they have also severely impacted soil health, leading to the depletion of soil fertility, loss of essential nutrients, and diminished water-holding capacity^2^. Furthermore, the overuse of these chemicals contributes to environmental issues such as eutrophication and the decline of biodiversity.

Among the pesticides commonly employed in agriculture, dimethoate, an organophosphate, is used in abundance regardless of the environmental threat it bears. It poses significant risks to non-target organisms, including humans and aquatic life, causing genotoxicity and developmental issues^3^. Despite regulations aimed at limiting the use of such xenobiotics, dimethoate remains prevalent due to economic pressures and a lack of awareness regarding its environmental consequences^4,5^. Its persistence in groundwater and surface water, despite a half-life of 10–14 days^6^, raises serious concerns about its long-term ecological impact.

Sustainable agriculture emphasizes the improvement and maintenance of soil health, the production of high-quality crops, and the enhancement of socio-economic balances^7^. A promising approach involves leveraging the potential of soil microorganisms, often referred to as “natural soil engineers”^8^. These microbes play crucial roles in nutrient cycling, soil enrichment, and the bioremediation of contaminants. A number of studies have been conducted for the bio-degradation of dimethoate using microbial agents like *Kocuria turfanensis* isolated from soil^9^, *Bacillus licheniformis* and *Pseudomonas aeruginosa* discerned from fish intestines and water respectively^10^, *Pseudomonas kilonensis*^11^ etc.

The rhizosphere is an important zone of the soil where a lot of biochemical exchange takes place between plants and soil microbiota. For this reason, in this zone, the microbial colonization rate is quite higher than the rest of the bulk soil^12^. A typical group of bacteria is often found associating with roots and rhizosphere soil that promotes growth of the plants and is known as plant growth promoting bacteria (PGPB). Beside this, it also plays an exclusive role in nutrient cycling, soil enrichment, breakdown of complex toxic compounds like pesticides, and combating environmental stress like high salinity, high pH, drought, or other extreme conditions. PGPBs can also produce various metabolites like siderophores, antibiotics, enzymes, Volatile Organic Compounds (VOC) that show antagonistic effects on phytopathogens^13^. An efficient PGPB to be applied in agriculture should be able to colonize plant roots and phyllosphere and also develop protective barriers like biofilm or microcolony formation^8^. Biofilm is an important protection strategy in microorganisms in various ways, like mechanical stability, nutrient accumulation, gene exchange, survival in stress conditions, and tolerance to toxic agents. Microorganisms release extracellular polymeric substances (EPS), facilitating attachment to biotic or abiotic surfaces, and gradually form an extracellular matrix (ECM) by embedding the bacterial cells^14^. *Bacillus*, *Rhizobium*, *Rhodopseudomonas and Agrobacterium* from the roots of *Larrea divaricata*^15^, *Bacilli* isolated from native maize landraces as seed-endophytic^16^, *Bacillus vallismortis* from the tea rhizosphere^17^ are few evidences of biofilm forming PGPB, providing protection to the bacteria as well as promoting distinctive plant growth. Biofilm often works as a stress response strategy for bacteria exposed to pesticides. However, obtaining an eligible PGPB candidate with biofilm forming ability is quite challenging, as the survival of targeted PGPBs often becomes questionable in in-vitro, when exposed to agrochemicals or fluctuations in environmental conditions. Biofilm-forming bacteria indigenous to organophosphate contaminated sites metabolically utilize and gain resistance to the pesticides and develop efficient mechanisms for their biodegradation. Rhizospheric soil collected at contaminated sites provides a superior source of microorganisms having the potential to degrade pollutants^18^.

This study aims to isolate and characterize bacteria from rhizospheric soil contaminated with dimethoate, focusing on their pesticide-degrading capabilities and plant growth-promoting traits. The novelty of this research lies in its comprehensive approach to identifying bacterial isolates that not only effectively degrade the organophosphate dimethoate but also possess strong biofilm formation abilities. This dual functionality is crucial for their resilience in field conditions, where they must endure environmental stressors while promoting plant growth. Moreover, the study will analyze crude metabolite extracts to identify and elucidate the metabolic pathway, evaluate the safety and viability of degraded by-products, as well as the suitability of selected microbial candidates. Addressing a significant research gap, this work seeks to identify bacteria that possess a unique combination of pesticide degradation, biofilm formation, and plant growth promotion. The findings of this work could lead to the development of eco-friendly strategies that mitigate the impacts of agrochemical pollution while improving soil health and crop productivity.

## Materials and methods

### Study area and soil sample collection

A sugarcane cultivation field in Sevur, Vellore district, Tamil Nadu, India (12.9649° N, 79.1831° E), which had a history of organophosphate pesticide dimethoate exposure for over 10 years was selected for soil sampling. Collection of rhizospheric soil was done from uprooted sugarcane plants of 15 random areas in the field. By tapping on roots, the adhered soils were collected in a sterile plastic bag, which was then taken to the laboratory. The soil samples were mixed thoroughly, and lumps were broken down into powder. The soil was tested for its physiological and chemical properties. Fresh soil samples were used for the experiments conducted.

### Isolation and screening of potential dimethoate degrading bacteria

The soil samples brought into the laboratory were artificially enriched with technical grade dimethoate (30% emulsifiable concentrate (EC), Rallis India Ltd., Mumbai, India.), according to the procedure described by Naphade et al. with few modifications^19^. Under aseptic conditions, in a 150 ml conical flask, 1 g of soil sample was added to 50 ml sterilized nutrient broth, and a 50 ppm concentration of dimethoate was added. The flask was kept in a rotatory shaker (180 rpm) for 5 days at room temperature. At day 5, 1 ml of stock (spiked soil sample) was taken, serial dilutions were performed (10^−1^ to 10^−10^) and spread plated on nutrient agar (pH 7, Hi-Media) [Peptone 5.000 g/L, Sodium chloride 5.000 g/L, HM peptone B# 1.500 g/L, Yeast extract 1.500 g/L, Agar 15.000 g/L]; incubated at room temperature (28–30 °C) for 24 h. Subsequent subcultures of individual colonies were performed to obtain and ensure pure cultures. The pure cultures obtained after isolation from rhizospheric soil samples, were further screened for potential dimethoate degrading strains. Onto selective mineral salt media (MSM, pH 7.0, Hi-Media) [NaCl 0.50 g/L, KH_2_PO_4_ 3.00 g/L, MgSO_4_ 0.12 g/L, CaCl_2_.2H_2_O 0.013 g/L, yeast 3.00 g/L, Na_2_HPO_4_ 6.00 g/L] supplemented with 150 ppm of dimethoate, the isolates were streaked. The plates were incubated for 24 h at 28–30 °C. The strains showing maximum viability were selected for further studies. The taxonomic characteristics and the colony features of the selected isolates were observed keenly. To detect the basic morphology and properties of the strain, biochemical tests and Gram staining were performed.

### Detection of dimethoate biodegradation

#### UV-spectrophotometer analysis of dimethoate biodegradation

The biodegradation of dimethoate in Mineral Salt Medium (MSM) was assessed using UV-spectrophotometric analysis. Cultures grown overnight in MSM media spiked with dimethoate, were centrifuged at 4000 g for 5 min at 4 °C. The pellet formed was washed with sterile 0.9% NaCl (saline) solution and resuspended in saline to achieve an optical density (OD) of 0.7. A 2% (v/v) aliquot of this seed culture was then inoculated into Erlenmeyer flasks containing 150 ml MSM media, supplemented with 150 ppm of dimethoate and adjusted to an initial pH of 7. The experimental setup was monitored for 5 days to track the degradation process of dimethoate by the isolate L.O. Uninoculated media with the identical parameters served as the control. At an interval of 24 h, 2 mL of broth sample was collected to measure cell density at OD 600. Following 5 days of incubation, the sample was centrifuged at 7000 g for 10 min to obtain the cell-free supernatant (CFS), which was then analysed by UV-spectrophotometry to quantify dimethoate degradation at 214 nm. The experiment was performed in triplicate to ensure accuracy and reproducibility. The % degradation was calculated by the formula:

$$\% {\text{ Degradation}}={\text{ }}\left[ {\left( {{{\text{A}}_{\text{i}}} - {{\text{A}}_{\text{f}}}} \right)/{{\text{A}}_{\text{i}}}} \right]{\text{ }} \times {\text{1}}00,$$

where A_i_ is the initial absorbance and A_f_ denotes the final absorbance.

#### Extraction of crude metabolites by liquid-liquid extraction and FTIR analysis

The crude metabolite extract of isolated strain L.O was subjected to Fourier transform infrared spectroscopy analysis (FTIR), to detect any alterations in chemical bonds or functional group modifications occurring, due to the metabolic activity of the isolate in the course of dimethoate degradation^20^. Liquid–liquid extraction was performed to obtain crude extract from the culture medium^21^. The isolate was cultured in a conical flask containing MSM broth supplemented with dimethoate on shaker for 5 days at 180 rpm, 28–30 °C. Uninoculated dimethoate supplemented media was used as a control (untreated). After 5 days, the broths were centrifuged at 6000 g for 10 min to obtain cell free supernatant (CFS). In a separating funnel, metabolites were extracted by mixing equal volumes (1:1) of supernatant with chloroform (solvent). The supernatant was thoroughly shaken with the solvent and allowed to stand for about 30 min to facilitate the complete separation of the chloroform layer from the aqueous phase. This crude chloroform extract was collected, dried to remove residual solvent and used for FTIR analysis. The FTIR analysis was conducted using the Nicolet iS50 instrument from Thermo Scientific, USA. The analysis covered a spectral range from 4000 cm^−1^ to 500 cm^−1^ with a resolution of 4 cm^−1^. Peaks in the FTIR spectrum are developed based on the percentage transmission of IR light at specific frequencies. Comparative changes of chemical bonds and functional groups associated with the peaks were observed for detecting bio-degradation of the dimethoate (untreated) and the treated sample. The data were identified using the frequency tables provided by Nandiyanto et al.^22^.

#### Identification of secondary metabolites and analysis of probable dimethoate biodegradation pathway

The crude chloroform extract containing the secondary metabolites produced by the bacterial isolate was analysed using GCMS^23^. GC–MS (VIT-SIF Lab, Division of Chemistry for NMR and GC-MS Analysis). The Clarus 680, Perkin Elmer GCMS instrument was specified with an Elite-5MS column with 30.0 m length, 250 μm film thickness and 0.25 mm internal diameter. To separate the components, helium was employed as carrier gas at a constant flow rate of 1 mL/min. The chromatographic run was executed at 260 °C injector temperature. 1 µL of the extracted residue was injected into the instrument for 2 min with the initial oven temperature at 60 °C, followed by 300 °C at the rate of 10 °C/min, and 300 °C was possessed for 6 min. Total GC running time was 64 min. The obtained spectra of the metabolite components were compared with the database of known components’ spectra correlating the GC-MS NIST (2008) library using Tuerbomass Version 5.4.2 software. To propose a prospective biodegradation pathway for dimethoate, GC-MS data were analyzed to identify degradation products. The PathPred pathway prediction server from GenomeNet was used to predict metabolic pathways based on these compounds, facilitating the identification of intermediate metabolites and associated enzymes. Additionally, the KEGG (Kyoto Encyclopedia of Genes and Genomes) database was used to visualize the curated metabolic networks related to dimethoate degradation. The resulting pathway was adapted and redrawn from the findings of Sharma et al.^24,11^.

### Identification of bacterial isolate

To identify the potential pesticide degrading bacterial isolate L.O, 16 S rRNA sequencing was performed by the Sanger sequencing method using the ABI 3130xl platform. From cellular DNA, the 16 S rDNA gene was amplified by PCR using 16 S rDNA-specific primers (Forward: GGATGAGCCCGCGGCCTA, Reverse: CGGTGTGTACAAGGCCCGG)^25^. The polymerase chain reaction amplification was achieved in a final volume of 50 µl. The amplification reaction containing 144 ng of extracted DNA is used for amplification along with 10 pM of each primer, dNTPs 2.5 mM, 10×Taq DNA polymerase assay buffer and Taq DNA polymerase enzyme. It was run on an ABI3130 genetic analyzer. The amplification reaction was set for 30 cycles. PCR conditions were set to initial denaturation at 94 °C for 3 min, 32 cycles consisting of denaturation at 94 °C for 1 min, annealing at 50 °C for 1 min, extension at 72 °C for 1 min, and final elongation at 72 °C for 7 min. Using NCBI Blast^26^, the sequence similarity was analysed and compared with the reference strains from Genbank and the Ribosomal Database. Fastree software was used to construct the phylogenetic tree incorporating the neighbor-joining method^27^.

### Scanning electron microscopy (SEM) analysis

The impact of dimethoate on the cell surface morphology of the isolated strain LO was investigated using scanning electron microscopy (SEM)^28^. Cell culture grown overnight in MSM media, both with and without dimethoate treatment at log phase, was used for sample preparation. For SEM sample preparation, the bacterial biomass was fixed in a glutaraldehyde solution (4:1 ratio) to preserve cellular structures, followed by washing with phosphate-buffered saline (PBS) at a 1X concentration to remove excess fixative. A gradual dehydration process was further conducted using a series of ethanol solutions (20%, 40%, 60%, 80%, and 100%), ensuring the maintenance of cell morphology. The samples were subsequently set for SEM observation, operated at a voltage of 10 kV, and imaged at an 8KX magnification. Cultures grown without dimethoate treatment served as the control group, which allowed a comparative analysis of any morphological changes induced by the pesticide treatment. The SEM images were analysed to assess alterations in cell surface structures of the isolate, providing insights into the effects of dimethoate on the isolated strain.

### Characterizations of the isolates

#### Analysis of PGPR activity

The isolate was tested for plant growth promoting activity like phosphate solubilization activity and production of HCN, IAA, ammonia and siderophore as per the protocols described by Sharma et al.^29^. All the tests were performed in triplicate.

#### Phosphate solubilization

Phosphorus solubilizing activity of isolates was determined qualitatively according to the method stated by Nautiyal^30^. To perform the experiment, 2.5 µl of the isolate L. O (O.D. 600) was spread plated onto Pikovskaya’s agar medium [(g/L) Yeast extract 0.500, Dextrose 10.000, Calcium phosphate 5.000, Ammonium sulphate 0.500, Potassium chloride 0.200, Magnesium sulphate 0.100, Manganese sulphate 0.0001, Ferrous sulphate 0.0001, Agar 15.000] containing calcium triphosphate (0.5%) as the inorganic form of phosphate. The plates were incubated for 5 days at 28 °C. A transparent halo zone around the grown colonies indicates the phosphate solubilizing activity of the bacterial isolates.

##### Production of HCN

The targeted pesticide-tolerant bacterial isolate L.O was streaked onto nutrient agar plates supplemented with 4.4 g/L glycine. Filter paper discs, pre-treated with 0.5% picric acid were dissolved in 2% sodium carbonate, and placed inside the lid of each Petri dish. The dishes were then sealed and incubated for 5 days at 28 °C. A gradual colour change in the filter paper from deep yellow to orange and then to brown would indicate the production of hydrogen cyanide (HCN) by the target bacteria^29^.

##### Production of indole acetic acid (IAA)

The isolate L.O was inoculated in tryptophan (1–2%) supplemented nutrient broth. The flasks were kept on rotary shaker and incubated for 24 h at 28 °C. Cultures were centrifuged at 10,000 rpm for 15 min. The supernatant was taken and mixed with Salkowski’s reagent, and incubated at room temperature for 25 min. Appearance of the pink colour indicates production of Indole acetic acid (IAA)^29^.

##### Production of ammonia

The bacterial isolate L.O was cultured in peptone water for 4 days at 30 °C. 1 ml of Nessler’s reagent was added to the tube. Development of a dull yellow color indicates small amounts of ammonia production, and a deep yellow to brownish color indicates maximum ammonia production^29^.

##### Production of siderophore

Siderophore production of the isolate was detected by the chrome azurol S (CAS) assay^29^. Bacterial isolate grown on nutrient agar was spot inoculated on CAS agar [Agar 15 g/l, Chrome Azurol S 0.02 g/l (1% solution and used as 10 ml/l), nutrient broth 5 g/l, KCl 0.1 g/l, MgSO_4_·7 H_2_O 0.2 g/l, CaCl_2_·2 H_2_O 0.01 g/l, FeCl_2_·6 H_2_O 0.001 g/l] and incubated for 7 days at 28 ± 0.2 °C in a dark room. An orange zone around the isolate indicates positive siderophore production.

#### Detection of biofilm formation by the tube method

The isolate L.O was tested for biofilm forming ability by the tube method^31^. The strain was inoculated in a set up containing MSM with a 150 ppm dimethoate supplement. The culture was incubated for 5 days at room temperature. After incubation, the tube was poured out, washed with phosphate buffer saline (pH 7.3) and air dried. The tube was then stained with 0.1% crystal violet. Excess and residual stain was removed with deionized water and then dried inside out. Tubes containing only media and no inoculation were used as control. The control tube was used as a reference to assess the differences in biofilm formation of the sample tube. Visible purple film lining the walls and bottoms of the tubes indicates a positive indication of biofilm formation. The experiment was performed in triplicate.

### Ecotoxicity studies

#### Phytotoxicity analysis

The biodegraded metabolites were assessed for their toxicity compared to the parent compound dimethoate. Phytotoxicity tests were performed as per the method described by Sahoo et al.^32^. In this experiment, the phytotoxicity of metabolites obtained from dimethoate biodegradation in comparison to the parent compound was performed against mustard seeds (*Brassica juncea*) due to its widespread cultivation in India and rapid germination rate. The seeds were initially washed three times with distilled water to remove surface contaminants, followed by surface sterilization using 70% ethyl alcohol, and then rinsed again with distilled water. Experimental setups involved preparing cotton beds within Petri dishes, overlaid with Whatman No. 1 filter papers, which were subsequently moistened with sterile distilled water to create optimal germination conditions for the seeds. Minimal Salts Medium (MSM) solutions containing specific concentrations of dimethoate and crude extracts of biodegraded metabolites were added to individual Petri plates, while control plates consisted of MSM and tap water only. Ten surface-sterilized seeds were placed in each Petri dish, maintaining adequate spacing to prevent overlapping of roots. The Petri dishes were covered with aluminum foil to maintain humidity and shield the seeds from light, and incubated in the dark for 24 and 48 h. Following incubation, germination rates and root lengths were measured. Each treatment was replicated in triplicates to ensure statistical reliability, allowing for a comprehensive analysis of the effects of the biodegraded metabolites and dimethoate on seed germination and root development.

#### Microbial toxicity study

The microbial toxicity of dimethoate and its biodegraded products was assessed using the Agar well diffusion method^32^. In this study, lawn cultures were prepared using model organisms *Bacillus subtilis* and *Escherichia coli*, selected for their well-characterized genetics, role in soil health, resistance mechanisms and are representative of enteric bacteria that can be impacted by environmental pollutants, making them an ideal candidate for assessing the effects of agricultural chemicals like pesticides^33,34^ (Sturme et al. 2021; Abd El-Ghany et al. 2023). Wells were created in the agar plates, into which crude dimethoate and the metabolic extracts were introduced for testing against the target microorganisms. The plates were incubated at 37 °C for 24 h to facilitate microbial growth and interaction with the test compounds. Following incubation, the presence of any clear zones of inhibition around the wells, indicating the antimicrobial efficacy of the compounds tested was observed and noted. The experiment was conducted in triplicates to ensure the reproducibility and reliability of the results, providing a comprehensive insight of the microbial toxicity of both dimethoate and its degradation products.

## Results

### Isolation and screening of isolates

The initial screening generated morphologically distinct, mixed colonies on nutrient agar (NA) plates from spiked soil sample. The soil used had a pH of 7. In the control plate, no growth was seen. A potent dimethoate degrading strain L.O was selected based on the growth of the strain on MSM agar plates supplemented with 150 ppm dimethoate as the sole carbon source after incubation at 28–30 °C for 24 h. Subsequent subcultures were performed to obtain pure cultures. The isolated bacteria under investigation demonstrated several distinguishing characteristics relevant to its identification and ecological contribution. Colonies are opaque, glossy, and orange in colour, indicating robust cell structure and pigment production capability. The organism is classified as Gram-positive, stipulating a thick peptidoglycan layer that may confer sustainability against environmental stresses. The negative result of spore stain, implies a possibility of alternative survival strategies. A positive Voges-Proskauer test reflects the acetoin production ability, indicating fermentative metabolism, while negative results for citrate utilization and indole production suggest restricted metabolic versatility. Additionally, the negative oxidase test and positive catalase test highlight specific metabolic pathways that facilitate the detoxification of hydrogen peroxide. The positive reaction to starch hydrolysis demonstrates its ability to utilize complex carbohydrates, highlighting its adaptability in diverse ecological niches. Details of the morphological and biochemical characters have been listed in Table 1.

**Table 1:** Morphological and biochemical characteristics of the strain L.O.

Characteristics	Colony morphology	Color	Shape	Gram stain	Spore stain	Voges-Proskauer	Citrate utilization	Indole production	Oxidase test	Catalase test	Starch Hydro-lysis
Result	Opaque, glossy	Orange	Rod	+	−	+	−	−	−	+	+

### Detection of dimethoate biodegradation

**Fig. 1:**
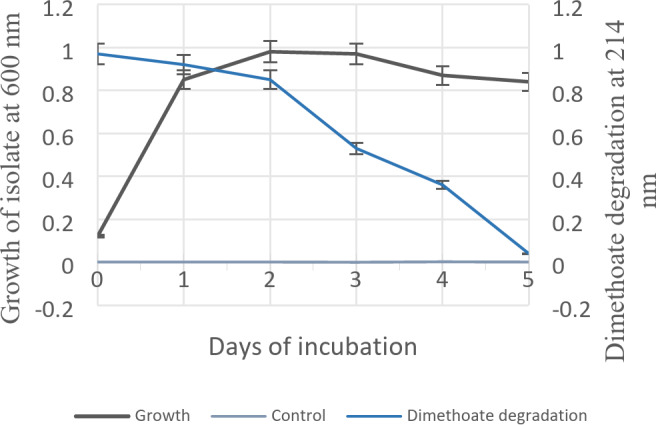
Graphical representation showing relation between cell density of isolate L.O and biodegradation of dimethoate.

The relation between the growth of isolate and the biodegradation of dimethoate pesticide has been demonstrated in Fig. 1 deducing the UV-spectroscopic readings. The analysis was carried out for five days. From Fig. 1, it is seen that, cell culture density (O.D_600_) is at its maximum after one day, and after three days bacterial concentration was decreasing marking the deterioration stage. Bio-degradation of dimethoate was achieved maximum after five days. The control showed no turbidity. The percentage of dimethoate degradation was enumerated as per the formula mentioned in Section "UV-spectrophotometer analysis of dimethoate biodegradation". The strain L.O. was found to be capable of degrading 95.87% of dimethoate in five days.

**Fig. 2:**
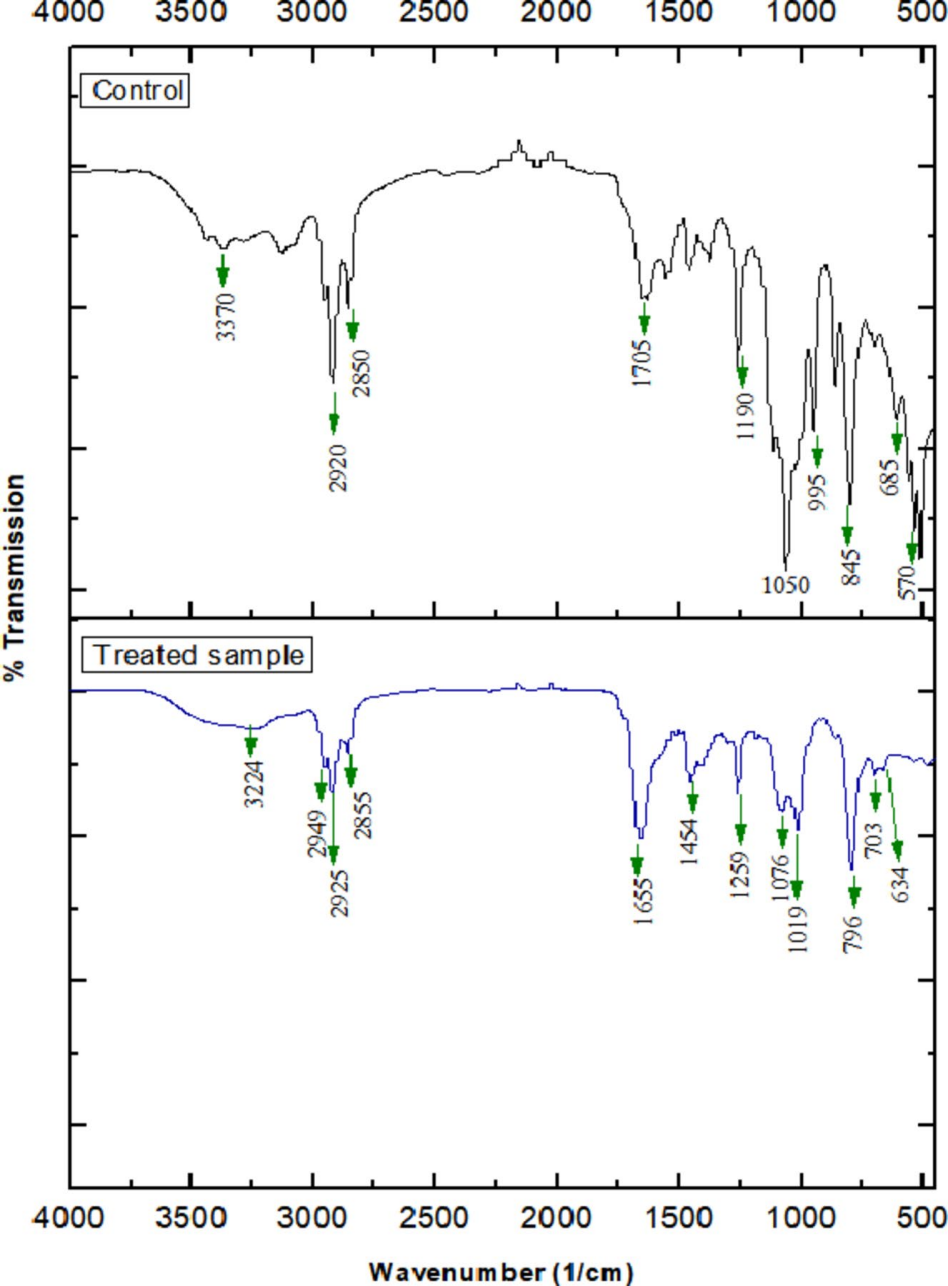
FTIR analysis of chloroform crude extract from L.O to detect biodegradation of dimethoate (up) control (parent compound), (down) treated sample.

The structural changes in dimethoate upon degradation by the isolate L.O were determined by FTIR-analysis. A lucid difference in spectrum was noticed between the untreated (control) and isolate-treated sample, indicating degradation of dimethoate, due to the activity of isolate by conformational changes or bond alterations. The spectrum has been shown in Fig. 2. In untreated dimethoate (control), peaks were noticed at 3370 cm^− 1^, 2920 cm^− 1^, 2850 cm^− 1^, 1705 cm^− 1^, 1190 cm^− 1^, 1050 cm^− 1^, 995 cm^− 1^, 845 cm^− 1^, 685 cm^− 1^ and 570 cm^− 1^. Whereas, for the treated sample, peaks were obtained at 3224 cm^− 1^, 2949 cm^− 1^, 2925 cm^− 1^, 2855 cm^− 1^, 1655 cm^− 1^, 1454 cm^− 1^, 1259 cm^− 1^, 1076 cm^− 1^, 1019 cm^− 1^, 796 cm^− 1^, 703 cm^− 1^ and 634 cm^− 1^. Peak pertaining to aliphatic POC phosphate (1050 cm^− 1)^ present in control, disappeared in the treated sample. There was a distinct reduction and peak shift in a range of 3000–3500 cm^− 1^, 2850 –2815 cm^− 1^, 710 –685 cm^− 1^ and 705–570 cm^− 1^. At 2935 –2915 cm^−1^ range, splitting of peaks was also observed in treated extract compared to untreated sample.

In the untreated sample, the presence of peaks at 3370 cm^−1^ and 1705 cm^−1^ corresponds to hydroxyl and carbonyl functional groups, respectively, which are typical in organophosphate compounds. The disappearance of the peak at 1050 cm^−1^, corresponding to aliphatic POC phosphate, in the treated sample is indicative of a successful degradation process, possibly reflecting the breakdown of the phosphonate group. Additionally, the shifts and reductions in peaks observed in the spectral regions around 3000–3500 cm^−1^ (aliphatic 2° amine stretch), 2850 –2815 cm^−1^ (methoxy CH stretch), and 710–570 cm^−1^ (disulfides) further reflect that the isolate not only degraded the organophosphate compound (dimethoate) but also modified its chemical environment, influencing its toxicity and environmental persistence.

The splitting of peaks in the 2935–2915 cm^−1^ (methylene CH stretch region) in the treated sample demonstrates changes in the molecular conformation, which may originate from interactions between the isolate and the dimethoate molecule during degradation. Overall, these spectral differences mark the effectiveness of isolate L.O. in degrading dimethoate, highlighting its potential application in bioremediation strategies targeting organophosphate pollutants.

### Identification of secondary metabolites and analysis of probable dimethoate biodegradation pathway

**Fig. 3:**
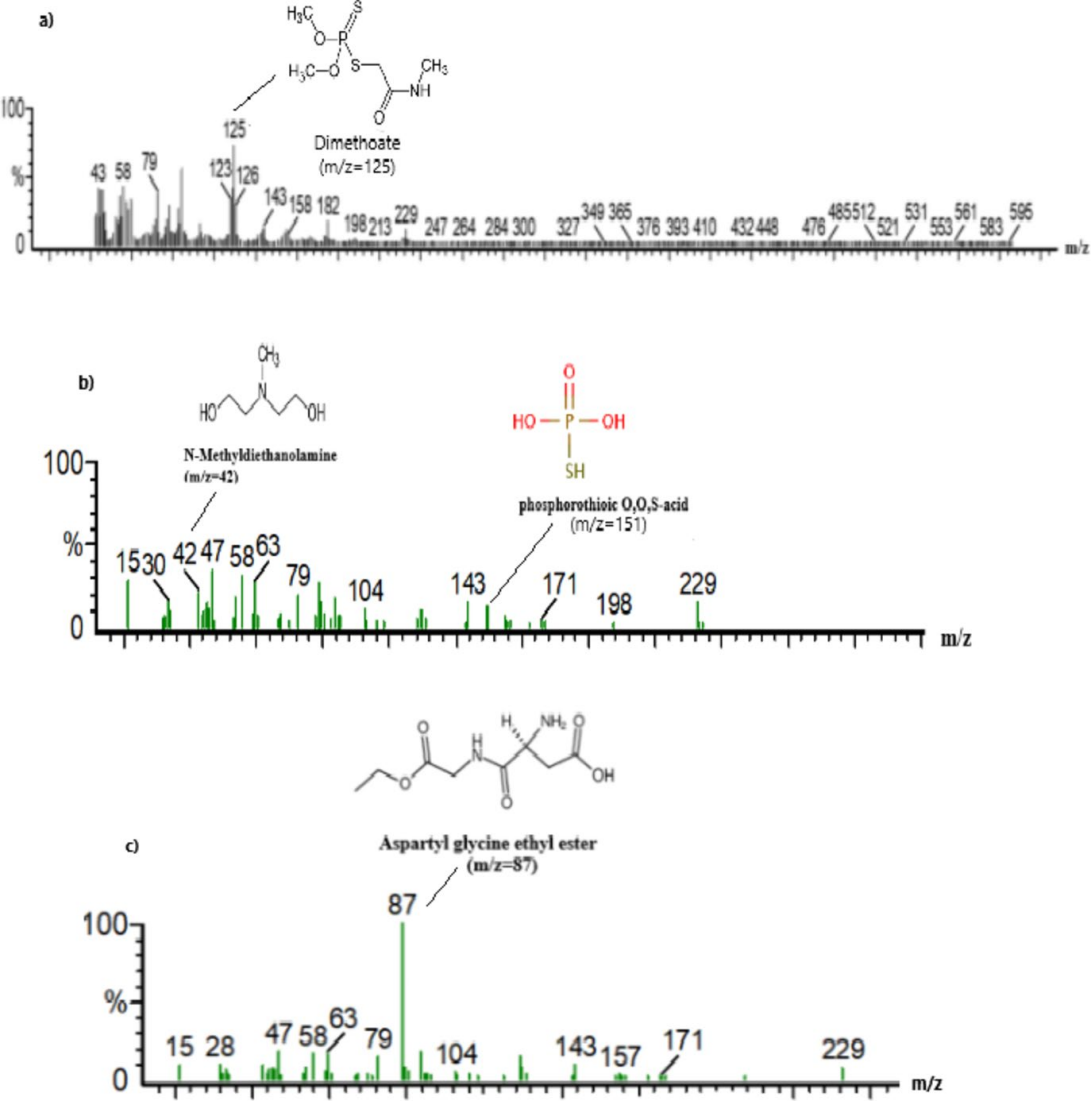
Mass spectrometry detection of dimethoate and its metabolites: a Dimethoate with *m*/*z =* 125, b methyl diethanol amine with *m*/*z* = 42, phosphorothioic acid with m/z = 151 and c aspartyl glycine ethyl ester with *m*/*z* = 87.

GC-MS analysis of by-products formed upon dimethoate bio-degradation imparts valuable insights into the chemical transformations by the metabolic activity of the isolate L.O (Fig. 3). The initial detection of dimethoate (m/z 125) in the untreated sample establishes its baseline presence. Following treatment, the identification of intermediate compounds such as methyl diethanol amine (m/z 42) and aspartyl glycine ethyl ester (m/z 87) justifies the event of hydrolytic and substitution reactions.

The hydrolysis of dimethoate possibly proceeds with the formation of methyl diethanol amine by cleaving the ester bond, releasing a methyl group, and incorporating ethanolamine. The formation of aspartyl glycine ethyl ester indicates enzymatic transformations utilizing these intermediate compounds in microbial metabolic activity, possibly denoting a pathway for assimilation of amino acids. The presence of phosphorothioic O, O, S-acid (m/z 151) in the treated sample signifies complete degradation of the dimethoate structure, directing towards a possible successful breakdown of the phosphorothioate moiety. This transformation provides an insight that the isolate L.O. utilizes oxidative and hydrolytic mechanisms to nullify dimethoate toxicity, resulting in less harmful products.

**Fig. 4:**
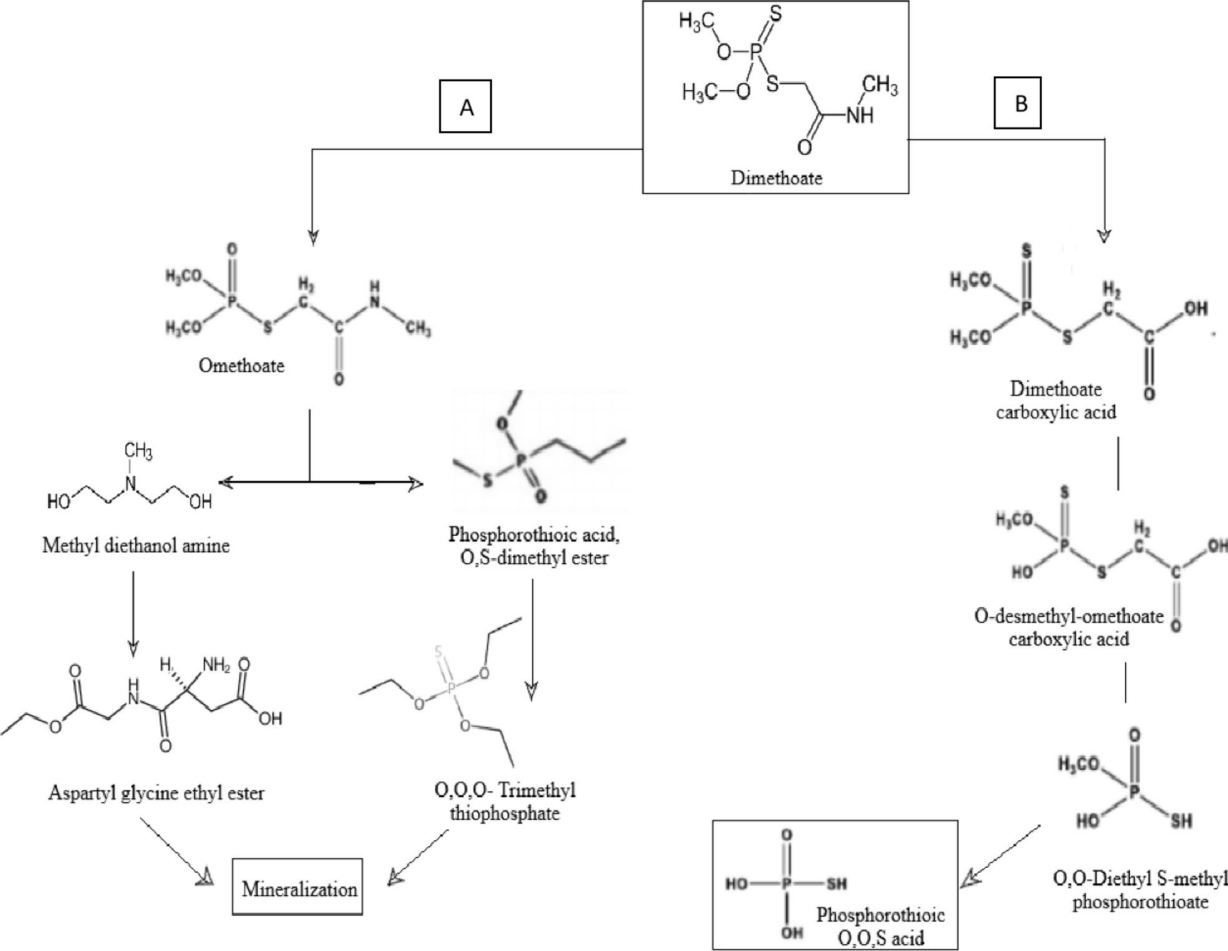
Proposed dimethoate bio-degradation pathway: (**A**) Omethoate pathway, (**B**) carboxylation pathway.

The proposed bio-conversion pathways (Fig. 4A, B) demonstrate two probable distinct routes of degradation. Figure 4A shows the conversion of dimethoate to omethoate via nucleophilic substitution, while Fig. 4B depicts a carboxylation route, indicating further degradation into non-toxic end products. These chemical pathways uphold the versatility of metabolic mechanisms of isolate L.O., emphasizing its potentiality in dimethoate detoxification strategies.

### Identification of bacterial isolate

**Fig. 5:**
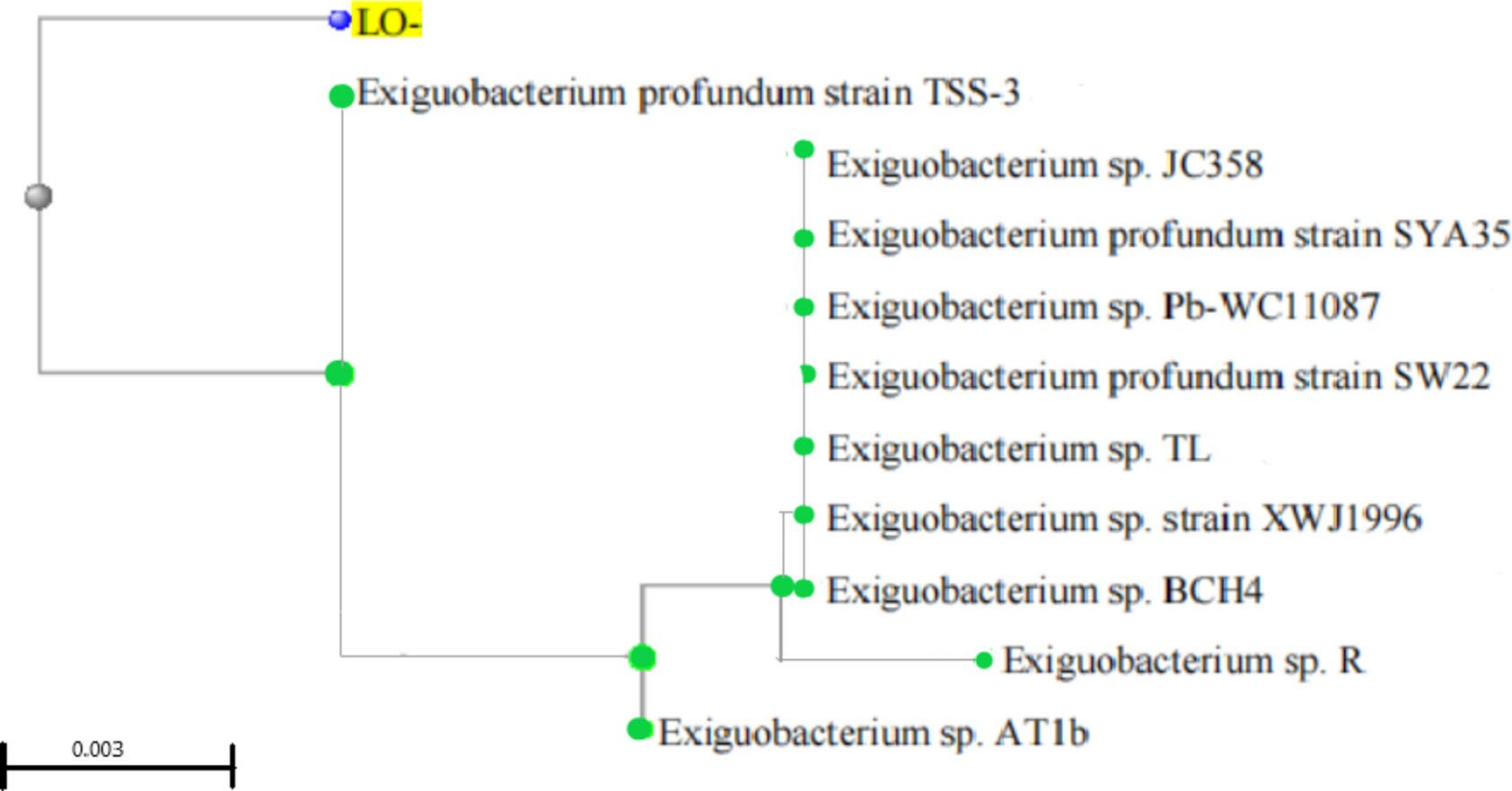
Phylogenetic tree based on 16 S rRNA gene nucleotide sequences between *E. profundum* L.O (NCBI accession number, OR965293) and reference sequences retrieved from NCBI GenBank constructed by the neighbour joining method.

The molecular identification of the selected bacterial strain L.O was performed using 16 S rRNA sequence analysis. The reference sequences (FASTA) were retrieved using the Basic Local Alignment Search Tool (BLAST) against the NCBI database, and the phylogenetic tree was generated incorporating the neighbour joining method. A phylogenetic tree was generated based on the collection of the 16 S rRNA sequences of the related species acquired from NCBI along with the query. The tree was constructed using MEGA 7 software and the constructed tree was visualized in iTOL v6^35^. The similarity of the identified species is accounted by the closest match with a short genetic distance with the closest neighbor. The strain was identified as *Exiguobacterium profundum* with 100% similarity, and was submitted to NCBI against accession number, OR965293 (Fig. 5).

### Scanning electron microscopy (SEM) analysis

**Fig. 6:**
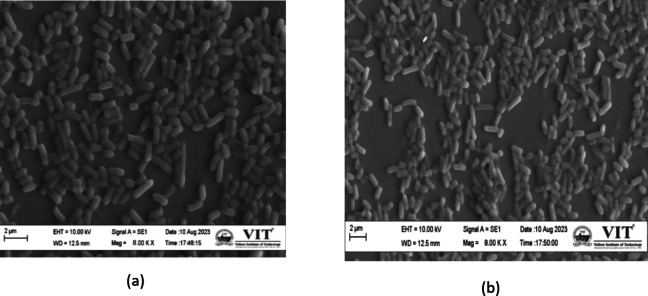
Scanning electron images of isolate L.O grown in dimethoate supplemented media. (**a**) Dimethoate-untreated culture, (**b**) dimethoate-treated culture at 8 KX magnification.

Comparative scanning electron microscopy (SEM) images of dimethoate-treated and untreated cultures of isolate L.O. (Fig. 6) at 8 kX magnification exhibits that the strain conserves the rod-shaped morphology with no prominent structural changes in cell surface post-dimethoate exposure. This structural integrity of the isolate L.O. signifies exclusive adaptation to the toxic impacts of dimethoate, suggesting effective cellular mechanisms like sophisticated cell wall structures or the presence of efflux pumps for detoxification or tolerance of the organophosphate pesticide. Such features are important for survival in contaminated environments and may enhance the efficiency of the isolate in bioremediation targeting organophosphate pollutants.

### Characterizations of the isolates

#### Analysis of PGPR activity

The isolate L.O. was tested for plant growth-promoting traits, including phosphate solubilization, hydrogen cyanide (HCN) production, indole-3-acetic acid (IAA) synthesis, ammonia production, and siderophore production. The results were found to be positive for HCN production, IAA synthesis, and ammonia production, while no siderophore formation was observed (Table 2).

The production of HCN and IAA, a key auxin, plays a crucial role in root development and overall growth of plants, suggesting that isolate L.O. may positively influence plant health through hormonal regulation. Positive indication of ammonia production further denotes increased nitrogen availability, supporting better nutrient uptake and growth of plants.

**Fig. 7:**
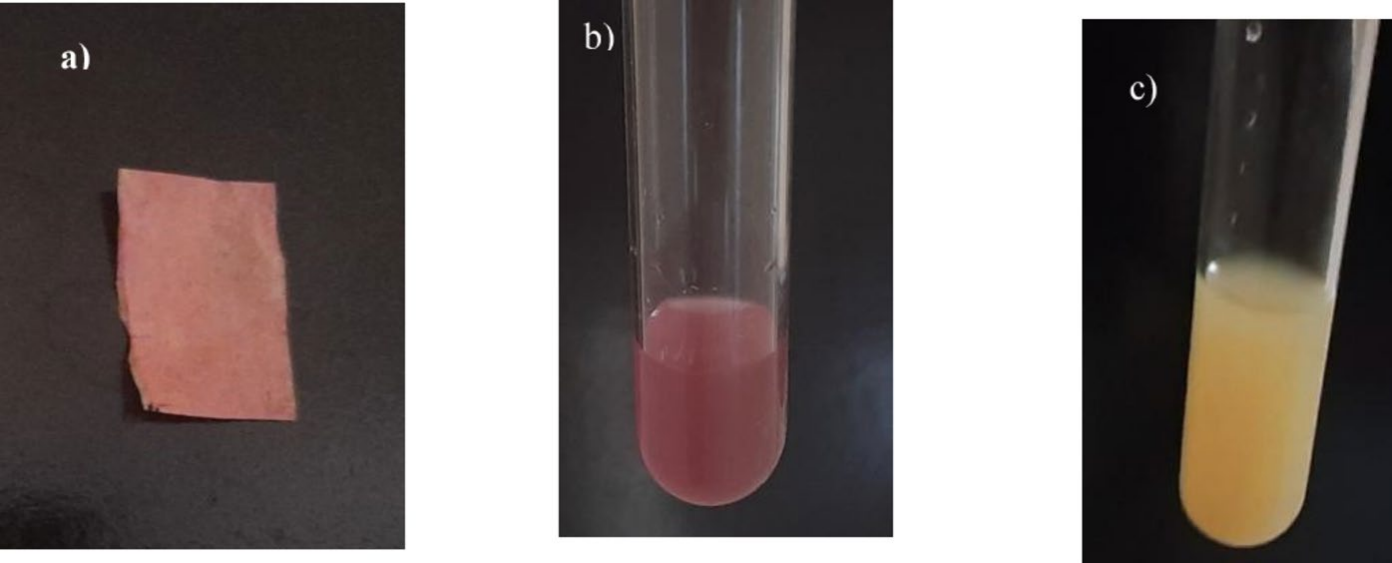
Analysis of PGP characters in selected isolates (**a**) color changes in filter papers due to HCN production by isolates, (**b**) test tubes indicating IAA production with pink colour formation, (**c**) isolates tested for ammonia production showing yellow color.

The lack of siderophore production may constrain the ability of the isolate to sequester iron in iron-deficient soils; however, the positive traits exhibited suggest that isolate L.O. can still make a significant contribution to plant enhancement through its other mechanistic approaches. Overall, these findings highlight the promising potential of the isolated L.O. as a beneficial microbial inoculant in agricultural developments targeting improved crop quality. The results of the PGPR activities shown by the isolates are demonstrated in Fig. 7.

**Table 2:** Plant-growth promoting traits shown by isolate L.O.

Isolate	Phosphate solubilzing activity	HCN production	IAA production	Ammonia production	Siderophore
L.O	−	+++	++	++	−

−Undetected, +low, ++medium, +++high.

#### Detection of biofilm formation by the tube method

**Fig. 8:**
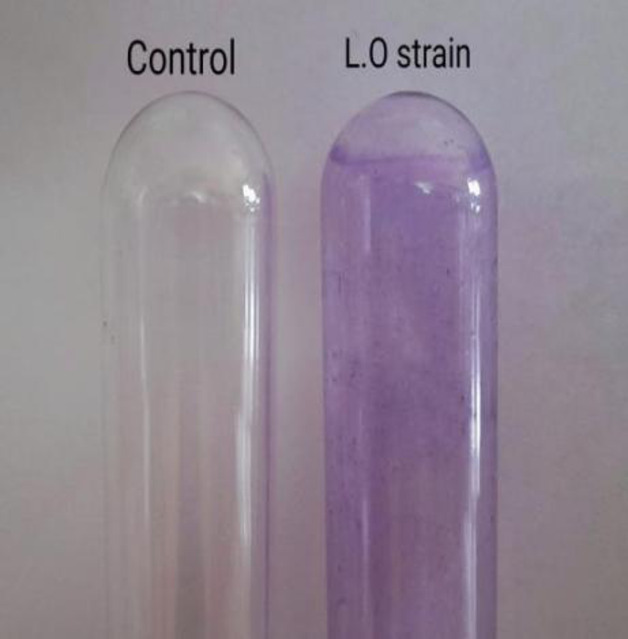
Tube showing purple coloration indicating biofilm formation by the isolates L.O. Control showed no coloration.

The strain L.O were checked for biofilm forming ability using tube method, based on the deposition of crystal violet on biofilm formed in the inner surface of tubes. The results indicated that strain L.O. adhered to the surfaces and generated biofilm. The tube appeared purple due to the retained crystal violet stain on the sides and bottom of the tubes, indicating biomass accumulation and effective adhesion properties of the strain. In contrast, the control showed no such purple coloration. The visual differences have been demonstrated in Fig. 8. The observation of the experiment establishes the potential of strain L.O. as a useful microorganism in agricultural settings, where biofilm formation may enhance its survival in field conditions.

### Ecotoxicity studies

#### Phytotoxicity analysis

**Fig. 9:**
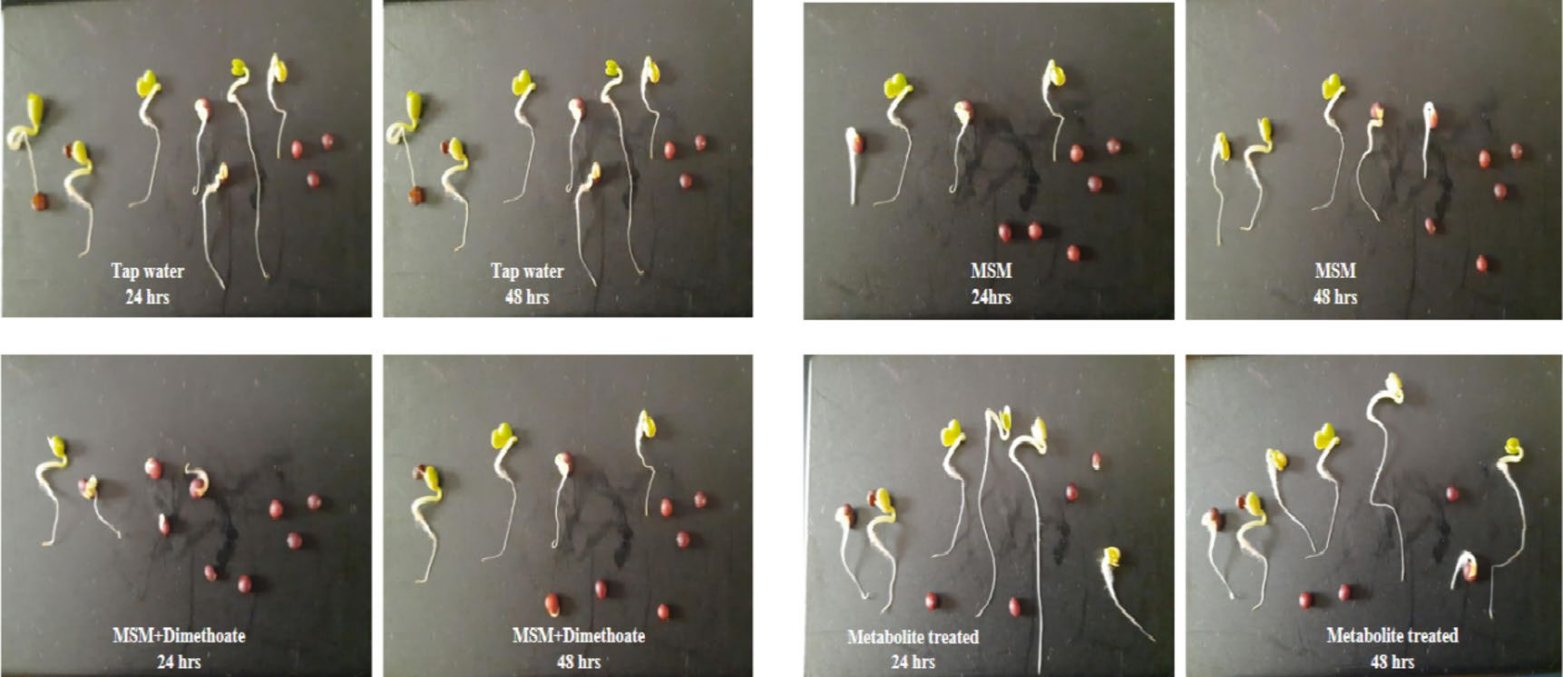
Phytotoxicity effect of various treatments in seed germination and root elongation.

**Fig. 10:**
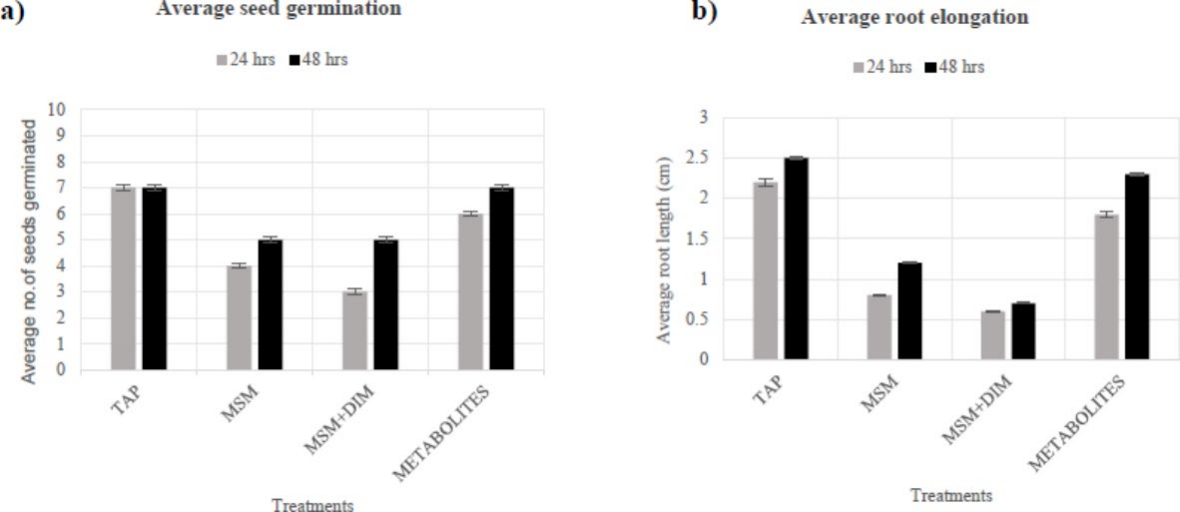
Phytotoxicity effect of pure dimethoate MSM + Dimethoate and its degraded products (metabolites) on (**a**) average no. of seed germination, (**b**) average root elongation in mustard seeds.

The toxicity of secondary metabolites resulting from the bacterial biodegradation of dimethoate was evaluated in mustard seeds through phytotoxicity to assess their suitability for environmental application. The results have been represented in Figs. 9 and 10, illustrating that average seed germination took almost 24 h for all the treatments. It was observed that MSM and dimethoate treated seeds showed less germination in 24 h as compared to control (Tap water). In contrast, a nominal difference in germination patterns between the control and metabolite-treated seeds was seen.

Maximum root elongation was observed in seeds treated with the metabolite extract, suggesting a possibility of positive influence of the biodegraded products on the seeds, while dimethoate-treated seeds exhibited the least root growth. This finding proposes that the metabolites derived from the biodegradation of dimethoate are comparatively safer than the toxicity associated with the parent compound, making them more suitable for environmental applications.

#### Microbial toxicity study

**Fig. 11:**
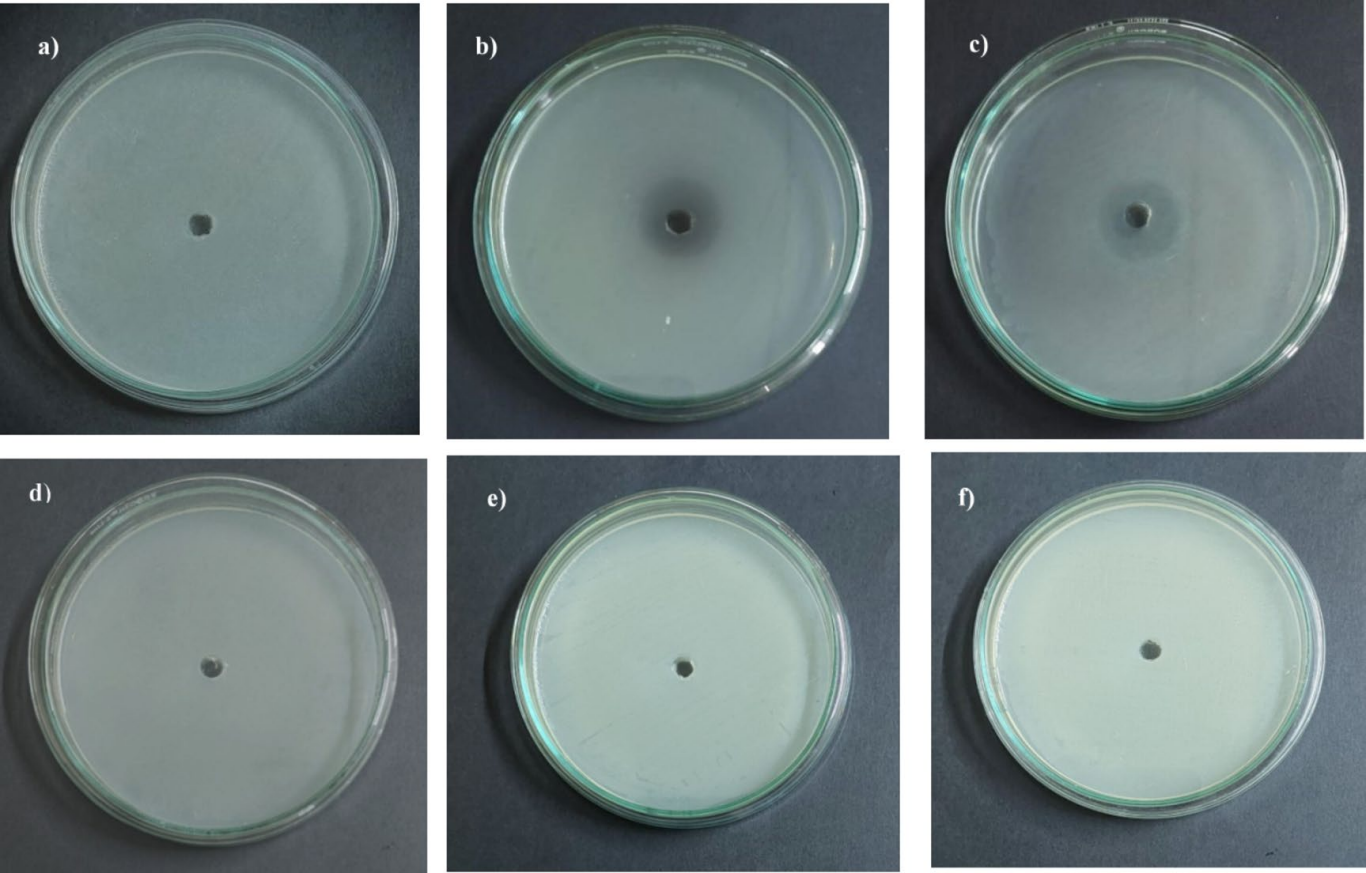
Microbial toxicity study with (**a–c**) dimethoate: (**a**) Control, (b) *B. subtilis*, (c) *E. coli*; (**d–f**) metabolite extract: (**d**) Control, (**e**) *B. subtilis*, (**f**) *E. coli*.

The toxicity of dimethoate and its degraded products was investigated in model microbes like *Bacillus subtilis* and *E. coli* by agar well diffusion method. Figure 11a–f shows the experimental results for microbial toxicity study. No zone of inhibition was observed for control (only MSM) and metabolite extract treated (degraded products) plates for all the microorganisms tested. However, clear zones were observed in the plates with crude dimethoate for both the tested bacteria. This result validates that the biodegradation products formed by the metabolic activity of L.O, in treatment of dimethoate, are less toxic than pure dimethoate towards the targeted microorganisms.

## Discussion

The isolation and characterization of strain L.O from dimethoate-contaminated rhizospheric soil portray its promising potential as a bioremediation agent. Cultured in minimal salt media supplemented with organophosphate dimethoate as the sole carbon source, this strain exhibited remarkable tolerance, effectively degrading 95.87% of the pesticide within five days. This finding is quite significant in context to the persistence of dimethoate in the environment and its associated risks to soil health and non-target organisms^24,36^. *Exiguobacterium* sp. have been reported to degrade organophosphate pesticides like acephate^37^, chlorpyrifos^36^, profenofos and malathion^38^, but there is no report found on dimethoate degradation for literature review so far.

Strain L.O was identified as *Exiguobacterium profundum* through 16 S rRNA sequencing, which are often considered extremophiles that thrive in diverse and often harsh environments. Earlier literature studies have documented the presence of *Exiguobacterium* species in various substrates, including mine soil^39^, plastic dumps^40^, saline sediments^41^, arsenic rich soil^42^, petroleum contaminated soil^43^, as well as Tibetan glaciers^44^. This adaptability suggests that strain L.O possesses resilient metabolic pathways that enable it to sustain under adverse conditions, enhancing its applicability in context to bioremediation. Significant alterations and shifts in peaks were observed in spectral data, indicating a possible biodegradation occurred due to activity of the isolate on dimethoate. A similar work has been reported by Silambarasan et al., where alterations in FTIR spectrum were observed between treated and untreated chlorpyrifos samples^28^. Analysing the GCMS data, a biodegradation pathway was elucidated^11,24^. From the data, it was illustrated that the biodegradation of dimethoate supposedly could have followed two routes, the dimethoate carboxylic acid pathway or the omethoate pathway, ending in phosphorothionic O, O, S acid and mineralization respectively which are probable safe end products that might possibly be released post bioremediation. High degradation efficiency of the isolate emphasizes its potential in mitigating the ecological impact of dimethoate contamination. Rapid degradation is crucial in bioremediation, as it reduces the duration of toxic exposure and minimizes the risk of harmful effects on soil, soil microbiome, water bodies, and plant life. Additionally, the morphological stability of strain L.O, evidenced by scanning electron microscopy (SEM), endorses its ability to maintain structural integrity in the presence of dimethoate. This contrasts with findings in other prior studies where pesticide exposure led to significant cell alterations, indicating that strain L.O exhibits effective protective mechanisms against pesticide-induced stress^45^.

Beyond its degradation capabilities, *Exiguobacterium profundum* exhibited various beneficial plant growth promoting (PGPR) properties such as hydrogen cyanide (HCN), indole-3-acetic acid (IAA), and ammonia, establishing L.O as a dual-function agent. These properties contribute to enhanced root development and nutrient uptake, simultaneously improving crop health in contaminated soils^41,43^. The ability to promote plant growth while degrading pollutants represents a promising approach for sustainable agricultural practices, improving soil qualities in affected regions. The capability of the strain to form biofilms under stress conditions further enhances its potential as an effective bioremediation candidate. Biofilms can provide protection against environmental stresses to the bacteria and increase the degradation efficiency of the contaminant^46^. Hypothesizing, if dimethoate serves as an inducer to biofilm formation, this could further enhance the functionality of the strain in contaminated environments.

However, survival of the potential bioremediation candidate is not the only concern. Sometimes, the by-products of biodegradation are more toxic than the parent compound^47^. Assessing the toxicity of the intermediate biodegradation product is an absolute necessity to prevent adverse effects of the treatment on the environment and ecosystem. Hence, ecotoxicity studies of the biodegradation by-products were conducted on mustard seeds and model bacteria like *B. subtilis* and *E. coli*, which were found to be less toxic than dimethoate, therefore can be considered safe. From the phytotoxicity test, an inhibitory effect was observed in seed germination and root elongation in dimethoate and MSM treated seeds. Pesticide toxicity and the strong ionic effect of MSM could be the possible reasons. This reverse effect was seen in metabolite treated mustard seeds and, thus, can be considered less toxic to the plants. In microbial toxicity studies, no zone of inhibition was observed in agar well diffusion assay with crude metabolic extract, which validates the safety of the strain *Exiguobacterium* sp. L.O and its dimethoate degradation by-products for the environment.

Strain L.O emerges as a promising candidate for the bioremediation of dimethoate-contaminated soils, offering remarkable advantages in degradation efficiency, morphological stability, and plant growth promotion. To maximize the potential of strain L.O, it is important to address the identified research gaps, including detailed mechanistic studies that elucidate the specific enzymatic reactions involved in the degradation process. Additionally, evaluating the long-term effects of the secondary metabolites on overall ecosystem health is necessary. This approach will help ensure that the bioremediation process does not inadvertently introduce new ecological risks, making the strain safer for application in agricultural systems and restoring contaminated ecosystems.

## Conclusion

This research presents a novel application of rhizospheric soil bacteria *Exiguobacterium sp*. (L.O), integrating the bioremediation of the organophosphate pesticide dimethoate with agricultural enhancement. This study contributes significant insights into the potential of the strain *Exiguobacterium profundum* as a bioremediation agent as well as a plant growth promoter, establishing the way for sustainable practices in managing pesticide pollution and enhancing agricultural productivity. This dual approach is crucial for managing the challenges posed by pesticide pollution in agricultural practices. Additionally, the capability of the strain to form biofilm enhances its resilience and survivability in contaminated environments, providing a strategic advantage under actual field conditions. To our knowledge, this study is the first to demonstrate the efficacy of this strain in the dimethoate degradation, as supported by an extensive review of the literature.

However, the study also highlights limitations that require further investigation, which include the need for long-term assessments of the effectiveness of the strain across diverse environmental conditions and its interactions and impact on the broader soil microbiome. While ecotoxicity studies on intermediate metabolites have been conducted for seed germination, future research should prioritize the evaluation of their effects on fully grown plants, assessing root and shoot growth, chlorophyll content, and flowering to obtain an insight into prolonged impacts on plant health and yield.

This research provides a significant understanding of the potential of *Exiguobacterium profundum* as a bioremediation agent and plant growth promoter, facilitating sustainable practices for managing pesticide pollution and enhancing agricultural productivity.

## Data Availability

The data that support the findings of this study are available in Google Scholar (https://scholar.google.com/). These data were derived from the following resources available in the public domain: Google Scholar (https://scholar.google.com/). The genomic data have been submitted to NCBI with acession number OR965293.

## References

[1] Hemathilake, D. M. K. S. & Gunathilake, D. M. C. C. Agricultural productivity and food supply to meet increased demands. In *Future Foods* 539–553 (Academic, 2022).

[2] Mitra, B. et al. Use of agrochemicals in agriculture: alarming issues and solutions. *Input Use Efficiency Food Environ. Secur.* 85–122 (2021).

[3] Silva, M. S. et al. Dimethoate induces genotoxicity as a result of oxidative stress: in vivo and in vitro studies. *Environ. Sci. Pollut. Res.* **28**(32), 43274–43286. 10.1007/s11356-021-15090-z (2021). 10.1007/s11356-021-15090-z 34189686

[4] Piwowarska, D. & Kiedrzyńska, E. Xenobiotics as a contemporary threat to surface waters. *Ecohydrol. Hydrobiol.* **22**(2), 337–354 (2022).

[5] Sharma, A. et al. Global trends in pesticides: A looming threat and viable alternatives. *Ecotoxicol. Environ. Saf.* **201**, 110812 (2020). 10.1016/j.ecoenv.2020.110812 32512419

[6] Van Scoy, A., Pennell, A. & Zhang, X. Environmental fate and toxicology of dimethoate. *Rev. Environ. Contam. Toxicol.* **237**, 53–70 (2016). 26613988 10.1007/978-3-319-23573-8_3

[7] Farooq, M., Rehman, A. & Pisante, M. Sustainable agriculture and food security. In *Innov. Sustain. Agric.* 3–24 (2019).

[8] Ajijah, N., Fiodor, A., Pandey, A. K., Rana, A. & Pranaw, K. Plant growth-promoting bacteria (PGPB) with biofilm-forming ability: a multifaceted agent for sustainable agriculture. *Diversity* **15**(1), 112 (2023).

[9] Barot, J. & Chaudhari, K. Analysis of dimethoate degradation by *Kocuria turfanensis* using GC–MS. *Asian J. Microbiol. Biotechnol. Environ. Sci.* **22**, 107–110. 10.1016/j.btre.2021.e00679 (2020).

[10] DebMandal, M., Mandal, S., Pal, N. K. & Aich, A. Potential metabolites of dimethoate produced by bacterial degradation. *World J. Microbiol. Biotechnol.* **24**, 69–72 (2008).

[11] Yasmin, A., Ambreen, S. & Shabir, S. Biotransformation of dimethoate into novel metabolites by bacterial isolate *Pseudomonas kilonensis* MB490. *J. Environ. Sci. Health B* **57**(1), 13–22. 10.1080/03601234.2021.2017723 (2022). 34978268 10.1080/03601234.2021.2017723

[12] Liu, S. et al. Nutrients in the rhizosphere: A meta-analysis of content, availability, and influencing factors. *Sci. Total Environ.* **826**, 153908 (2022). 10.1016/j.scitotenv.2022.153908 35183641

[13] Orozco-Mosqueda, M. D. C. et al. Plant growth-promoting bacteria as bioinoculants: attributes and challenges for sustainable crop improvement. *Agronomy* **11**(6), 1167 (2021).

[14] Mahto, K. U., Priyadarshanee, M., Samantaray, D. P. & Das, S. Bacterial biofilm and extracellular polymeric substances in the treatment of environmental pollutants: beyond the protective role in survivability. *J. Clean. Prod.* **379**, 134759 (2022).

[15] Fernández, L. et al. Isolation and characterization of plant growth promoting bacteria (PGPB) from *Larrea divaricata* Cav., with potential use in phytoremediation of mining soils. *Environ. Sustain.* **6**(2), 271–281 (2023).

[16] Gastélum, G., Ángeles-Morales, A., Arellano-Wattenbarger, G., Guevara-Hernandez, E. & Rocha, J. Biofilm formation and maize root-colonization of seed-endophytic Bacilli isolated from native maize landraces. *Appl. Soil Ecol.* **199**, 105390 (2024).

[17] Maitra, D., Roy, B., Chandra, A., Choudhury, S. S. & Mitra, A. K. Biofilm producing *Bacillus vallismortis* TR01K from tea rhizosphere acting as plant growth promoting agent. *Biocatal. Agric. Biotechnol.* **45**, 102507 (2022).

[18] Liaqat, I. et al. In vitro biofilm-mediated biodegradation of pesticides and dye-contaminated effluents using bacterial biofilms. *Microorganisms* **11**(9), 2163. 10.3390/microorganisms11092163 (2023). 37764007 10.3390/microorganisms11092163 PMC10535849

[19] Naphade, S. R., Durve, A. A., Bhot, M., Varghese, J. & Chandra, N. Isolation, characterization and identification of pesticide tolerating bacteria from garden soil. *Eur. J. Exp. Biol.* **2**(5), 1943–1951 (2012).

[20] Sriram, K. P., Mangrolia, U. & Osborne, W. J. Isolation and characterization of culturable indigenous endophytic bacteria in the tender coconut. *Food Biotechnol.* **34**(3), 228–242. 10.1080/08905436.2020.1789872 (2020).

[21] Reddy, S. & Osborne, J. W. Biodegradation and biosorption of reactive red 120 dye by immobilized *Pseudomonas guariconensis*: kinetic and toxicity study. *Water Environ. Res.* **92**(8), 1230–1241 (2020). 32150781 10.1002/wer.1319

[22] Nandiyanto, A. B. D., Oktiani, R. & Ragadhita, R. How to read and interpret FTIR spectroscope of organic material. *Indones. J. Sci. Technol.* **4**(1), 97–118 (2019).

[23] Malla, M. A. et al. Optimization and elucidation of organophosphorus and pyrethroid degradation pathways by a novel bacterial consortium C3 using RSM and GC-MS-based metabolomics. *J. Taiwan Inst. Chem. Eng.* **144**, 104744. 10.1016/j.jtice.2023.104744 (2023).

[24] Ahmad, S. et al. Dimethoate residues in Pakistan and mitigation strategies through microbial degradation: a review. *Environ. Sci. Pollut. Res.* **29**(34), 51367–51383 (2022). 10.1007/s11356-022-20933-4 35616845

[25] Wiley, E. O., Brooks, D. R., Seigel-Causey, D. & Funk, V. A. *The Compleat Cladist: A Primer of Phylogenetic Procedures* (Natural History Museum, University of Kansas, 1991).

[26] Stover, N. A. & Cavalcanti, A. R. Using NCBI BLAST. *Curr. Protoc. Essent. Lab. Tech.* **14**(1), 11 (2017).

[27] Price, M. N., Dehal, P. S. & Arkin, A. P. FastTree 2—approximately maximum-likelihood trees for large alignments. *PLoS ONE* **5**(3), e9490 (2010). 20224823 10.1371/journal.pone.0009490 PMC2835736

[28] Silambarasan, S. & Abraham, J. Ecofriendly method for bioremediation of chlorpyrifos from agricultural soil by novel fungus *Aspergillus terreus* JAS1. *Water Air Soil. Pollut.* **224**, 1369 (2013).

[29] Sharma, A., Dev, K., Sourirajan, A. & Choudhary, M. Isolation and characterization of salt-tolerant bacteria with plant growth-promoting activities from saline agricultural fields of Haryana, India. *J. Genet. Eng. Biotechnol.* **19**(1), 99 (2021). 10.1186/s43141-021-00186-3 PMC8239113 34181159

[30] Nautiyal, C. S. An efficient microbiological growth medium for screening phosphorus solubilizing microorganisms. *FEMS Microbiol. Lett.* **170**, 2017–2021 (1999). 10.1111/j.1574-6968.1999.tb13383.x 9919677

[31] Al-Ani, A. G., Younis, K. M. & Al-Taee, S. M. Isolation and molecular identification of *Exiguobacterium profundum* from drain water and study of some physiological properties (2022).

[32] Sahoo, B., Ningthoujam, R. & Chaudhuri, S. Isolation and characterization of a lindane degrading bacteria Paracoccus sp. NITDBR1 and evaluation of its plant growth promoting traits. *Int. Microbiol.* **22**, 155–167 (2019). 30810939 10.1007/s10123-018-00037-1

[33] Sturme, M. H. et al. *Bacillus subtilis*: a model organism for studying microbial communities. *Microb. Ecol.* **82**, 123–134 (2021).

[34] Abd El-Ghany, S. M. et al. *Escherichia coli*: clinical significance and antibiotic resistance. *Antibiotics* **12**(3), 439 (2023).36978306

[35] Letunic, I. & Bork, P. Interactive tree of life (iTOL) v6: recent updates to the phylogenetic tree display and annotation tool. *Nucleic Acids Res.* **1**, 268 (2024). 10.1093/nar/gkae268 PMC11223838 38613393

[36] Lian, L., Xing, Y., Zhang, N. & Jiang, B. Identification of chlorpyrifos-degrading microorganisms in farmland soils via cultivation-independent and-dependent approaches. *Environ. Sci. Process. Impacts* **24**(7), 1050–1059 (2022). 35674203 10.1039/d2em00095d

[37] Lin, Z. et al. Novel pathway of acephate degradation by the microbial consortium ZQ01 and its potential for environmental bioremediation. *J. Hazard. Mater.* **426**, 127841 (2022). 10.1016/j.jhazmat.2021.127841 34844804

[38] Isworo, S. & Oetari, P. S. The chemical compounds from degradation of profenofos and malathion by indigenous bacterial consortium. *J. Pure Appl. Microbiol.* **15**(2), 897–915 (2021).

[39] Das, S. et al. Reduction of hexavalent chromium by *Exiguobacterium mexicanum* isolated from chromite mines soil. *Chemosphere* **282**, 131135 (2021). 10.1016/j.chemosphere.2021.131135 34470171

[40] Maroof, L., Khan, I., Hassan, H., Azam, S. & Khan, W. Microbial degradation of low density polyethylene by Exiguobacterium sp. strain LM-IK2 isolated from plastic dumped soil. *World J. Microbiol. Biotechnol.* **38**(11), 197 (2022). 10.1007/s11274-022-03389-z 35989357

[41] Barghoth, M. G., Desouky, S. E., Radwan, A. A., Shah, M. P. & Salem, S. S. Characterizations of highly efficient moderately halophilic toluene degrading *Exiguobacterium mexicanum* M7 strain isolated from Egyptian saline sediments. *Biotechnol. Genet. Eng. Rev.* **1**, 1–19 (2023). 10.1080/02648725.2023.2184053 36861663

[42] Sengupta, D., Chakraborty, S., Choudhury, S. S., Ganguli, S. & Mitra, A. K. Isolation and identification of unique arsenotolerant *Exiguobacterium indicum* DSAM62 from arsenic rich environment. *Adv. Zool. Bot.* **8**(4), 298–325 (2020).

[43] Delegan, Y. et al. Characterization and genomic analysis of *Exiguobacterium alkaliphilum* B-3531D, an efficient crude oil degrading strain. *Biotechnol. Rep.* **32**, e00678 (2021). 10.1016/j.btre.2021.e00678 PMC8502702 34660213

[44] Tedesco, P. et al. Isolation and characterization of strain Exiguobacterium sp. KRL4, a producer of bioactive secondary metabolites from a Tibetan glacier. *Microorganisms* **9**(5), 890 (2021). 10.3390/microorganisms9050890 PMC8143284 33919419

[45] Shahid, M., Zaidi, A. & Saghir Khan, M. Modulations in growth, structure, cell viability and antioxidant enzyme of a nodule bacterium *Mesorhizobium ciceri* induced by pesticides. *Environ. Dev. Sustain.* **23**, 4103–4119. 10.1007/s10668-020-00758-2 (2021).

[46] Pavez, V. B. et al. Characterization of biofilm formation by Exiguobacterium strains in response to arsenic exposure. *Microbiol. Spectr.* **11**(6), e02657 (2023). 10.1128/spectrum.02657-23 PMC10714750 37819075

[47] Yigit, N. & Velioglu, Y. S. Effects of processing and storage on pesticide residues in foods. *Crit. Rev. Food Sci. Nutr.* **60**(21), 3622–3641 (2020). 31858819 10.1080/10408398.2019.1702501

